# GWAS and genomic prediction of milk urea nitrogen in Australian and New Zealand dairy cattle

**DOI:** 10.1186/s12711-022-00707-9

**Published:** 2022-02-19

**Authors:** Irene van den Berg, Phuong N. Ho, Tuan V. Nguyen, Mekonnen Haile-Mariam, Iona M. MacLeod, Phil R. Beatson, Erin O’Connor, Jennie E. Pryce

**Affiliations:** 1 Centre for AgriBioscience, Agriculture Victoria, 5 Ring Road, Bundoora, AgriBioVIC 3083 Australia; 2 CRV Ambreed, PO Box 176, Hamilton, New Zealand; 3 School of Applied Systems Biology, La Trobe University, Bundoora, VIC 3083 Australia

## Abstract

**Background:**

Urinary nitrogen leakage is an environmental concern in dairy cattle. Selection for reduced urinary nitrogen leakage may be done using indicator traits such as milk urea nitrogen (MUN). The result of a previous study indicated that the genetic correlation between MUN in Australia (AUS) and MUN in New Zealand (NZL) was only low to moderate (between 0.14 and 0.58). In this context, an alternative is to select sequence variants based on genome-wide association studies (GWAS) with a view to improve genomic prediction accuracies. A GWAS can also be used to detect quantitative trait loci (QTL) associated with MUN. Therefore, our objectives were to perform within-country GWAS and a meta-GWAS for MUN using records from up to 33,873 dairy cows and imputed whole-genome sequence data, to compare QTL detected in the GWAS for MUN in AUS and NZL, and to use sequence variants selected from the meta-GWAS to improve the prediction accuracy for MUN based on a joint AUS-NZL reference set.

**Results:**

Using the meta-GWAS, we detected 14 QTL for MUN, located on chromosomes 1, 6, 11, 14, 19, 22, 26 and the X chromosome. The three most significant QTL encompassed the casein genes on chromosome 6, *PAEP* on chromosome 11 and *DGAT1* on chromosome 14. We selected 50,000 sequence variants that had the same direction of effect for MUN in AUS and MUN in NZL and that were most significant in the meta-analysis for the GWAS. The selected sequence variants yielded a genetic correlation between MUN in AUS and MUN in NZL of 0.95 and substantially increased prediction accuracy in both countries.

**Conclusions:**

Our results demonstrate how the sharing of data between two countries can increase the power of a GWAS and increase the accuracy of genomic prediction using a multi-country reference population and sequence variants selected based on a meta-GWAS.

**Supplementary Information:**

The online version contains supplementary material available at 10.1186/s12711-022-00707-9.

## Background

Urinary nitrogen leakage is an environmental concern in dairy cattle [1]. While measuring urinary nitrogen leakage directly is challenging, milk urea nitrogen (MUN) can be used as an indicator trait [2, 3]. Since MUN can be predicted from a milk sample, MUN values can be obtained for all the cows that go through routine milk recording for which mid-infrared (MIR) spectral data are generated, thus for a substantial dataset. However, in some countries, such as Australia, milk recording companies have only recently invested in machines capable of generating MIR spectral data, so international collaboration is one way to test a larger (shared) dataset.

For many novel traits, international collaboration can help to increase the size of the reference population for genomic prediction and thereby to increase prediction accuracy [4]. In a recent study, we have shown that genetic correlations between MUN in Australia (AUS) and MUN in New Zealand (NZL) were low to moderate, ranging from 0.14 to 0.58 depending on the days in milk and breeds analysed [5]. We also found that heritabilities of MUN differed between AUS and NZL. However, correlations for some milk traits between AUS and NZL range from 0.60 to 0.83 [6], which imply a likely substantial gene×environment interaction (G×E) between traits measured in both countries. Thus, MUN in AUS is unlikely to be the same trait as MUN in NZL and the benefits of sharing a reference population may be limited. Other explanations could include differences in measuring equipment. Furthermore, MUN is highly influenced by factors such as feed intake [7], which is generally not routinely recorded, and thus not accounted for in the statistical model. In addition to the phenotypic noise, differences in breed composition may also contribute to the low genetic correlation between MUN in AUS and MUN in NZL. Generally, combining reference populations increases genomic prediction accuracy only when populations are highly related [8]. Haile-Mariam et al. [6] showed that for milk production traits, a joint AUS-NZL reference population increased prediction accuracies in NZL bulls by up to seven percentage points in reliability.

Several studies have shown how sequence variants selected from genome-wide association studies (GWAS) can be used to increase the accuracy of genomic prediction compared to the use of standard single nucleotide polymorphism (SNP) chips [9–11]. Sequence variants can be especially beneficial for across-breed genomic prediction, because variants that are closer to the causal mutations are more likely to be consistently linked to the causal mutations in all populations [10–12]. Thus, selecting sequence variants that are associated with MUN in both AUS and NZL may increase genomic prediction accuracies compared to using variants from standard SNP chips. In beef cattle, Porto-Neto et al. [10] showed that selecting variants with the same direction of effect in a within-breed GWAS, can result in a stronger genetic correlation between the populations and increase the prediction accuracy of multi-breed prediction. This approach could have applications for other traits, especially those for which reference populations are small, and could be of interest for both multi-country and multi-breed prediction.

GWAS for MUN may help to identify quantitative trait loci (QTL) in addition to aiding the selection of variants for genomic prediction. Several GWAS have reported QTL associated with MUN [13–17]. However, the size of the datasets used in these GWAS was small, limiting their detection power. Combining the AUS and NZL data for MUN would provide a comparatively large, powerful dataset to detect QTL associated with MUN.

Our objectives were (1) to perform a meta-GWAS for MUN using records from up to 33,874 dairy cows and imputed whole-genome sequence data, (2) to compare QTL detected in the GWAS for MUN in AUS and in NZL, and (3) to use sequence variants to improve the prediction accuracy for MUN based on a joint AUS-NZL reference set.

## Methods

### Phenotypes

Table 1 summarizes the number of phenotypes used for our analyses. We used the same dataset that is described in greater detail in van den Berg et al. [5]. Briefly, phenotypes for milk urea nitrogen concentration (MUN) were available for 18,120 AUS and 15,753 NZL cows. If a cow had multiple records available, we only used the earliest record. While the majority (12,660) of the AUS cows were Holsteins, the majority of the NZL cows (11,959) were crossbreds. In spite of the small number of records available for Australian Reds and Ayrshires, they were included in the dataset because some of the crossbreds (987 Australian cows and 1562 New Zealand cows) were part Australian Red and/or part Ayrshire.

**Table 1:** Number of records for milk urea nitrogen (n) per country and breed

Country	Breed	n
Australia	Holstein	12,660
	Jersey	1857
	Australian Red	95
	Ayrshire	12
	Crossbred	3496
	All	18,120
New Zealand	Holstein	2259
	Jersey	1524
	Ayrshire	11
	Crossbred	11,959
	All	15,753

MUN in AUS was derived from milk samples using commercial equations based on wet chemistry from Bentley instruments and MUN in NZL was derived using a FOSS MilkOscan FT + analyser (FOSS, Hillerød, Denmark).

### Reference and validation populations

Because of the heterogeneity of the crossbreds and the small number of Australian Reds and Ayrshires in the dataset, prediction accuracies were estimated for Holstein and Jersey only. All AUS and NZL Holstein and Jersey cows were randomly allocated to one of five validation folds. Validation was carried out within population: for each of the five folds, there were four validation sets of 2532 AUS Holstein, 371 AUS Jersey, 452 NZL Holstein and 305 NZL Jersey cows. For each of these validation folds, a mixed breed reference population was designed and included all the cows that were neither pure Holstein nor pure Jersey, plus the Holsteins and Jerseys that were not in the validation population.

### Genotypes

All animals used in our analyses were genotyped. We used SNP genotypes from the BovineHD Genotyping BeadChip (HD) as well as imputed whole-genome sequence data for our analyses. The NZL cows and most of the AUS cows were genotyped with low-density (~ 8500) SNP panels that had an overlap of approximately 7000 SNPs with the Illumina Bovine 50K panel. A small number of the AUS cows were genotyped on the Illumina Bovine 50K panel. All raw genotypes underwent quality control checks based on the GenCall (GC) score and were mapped to the ARS-UCD1.2 reference genome [18]. Any animal, or SNP with more than 10% of genotype calls with a GC score lower than 0.6 were discarded. Then, all remaining genotype calls with a GC score lower than 0.6 were set to missing, and the Fimpute v.3 software [19] was used with default settings to impute the sporadic missing genotypes. Low-density genotypes were first imputed to the BovineSNP50 BeadChip using a mixed breed (Holstein and Jersey) imputation reference set of 14,722 animals. These 50K genotypes were then imputed to the HD panel (~ 700K SNP) using a reference set of 2700 animals. Genotypes were imputed up to the HD panel using Fimpute v.3 and then converted to forward sequence format for imputation to whole-genome sequence. The imputation reference comprised 4190 *Bos taurus* cattle in Run8 of the 1000 Bulls Genome Project [20, 21]. Sequence imputation was implemented using the Minimac4 tool [22], and the Eagle v.2.4.1 software [23] was used to pre-phase the HD and reference sequences. The default Minimac settings were used, except that the window processing lengths were increased to –ChunkLengthMb 50 –ChunkOverlapMb 10, which allow to account for the longer haplotypes present in cattle genomes, compared to humans, and to ensure continuity in the overlapping segments. The Run8 reference sequences were processed following the 1000 Bull Genomes project pipeline (http://www.1000bullgenomes.com/) and the sporadic missing genotypes were imputed using the Beagle v4.1 package [24]. Prior to imputation, the reference sequence variants were further filtered: to retain only bi-allelic variants with an allele count of at least 4, and those with a Beagle R^2^ higher than 0.9. The pseudoautosomal region (PAR) of the X chromosome was not imputed. We used only sequence variants with a Minimac imputation R^2^ ≥ 0.4 and a minor allele frequency ≥ 0.005, which resulted in 16,824,460 variants that were used for the GWAS and genomic prediction.

### Principal component analysis

To account for breed effects, we used the first principal component (PC1) of a principal component analysis (PCA) performed with all AUS and NZL HD genotypes [5]. The PCA was undertaken using the genome-wide complex trait analysis (GCTA) software package [25].

### Genome-wide association analyses

Within-country GWAS were carried out using GCTA [25]. Phenotypes used for the GWAS were pre-corrected using fixed effects and covariates (test month, herd-year-season, days in milk, age, and PC1), and a genomic relationship matrix based on the HD genotypes was fitted to account for family structure. Because pedigree information was not available for the NZL cows, we used only genotypes for the analyses. A multi-country meta-GWAS was done using the weighted Z-score model implemented in the METAL software [26]. We used the weighted Z-scores model because in a previous study that compared different meta-analysis methods [27], the weighted Z-score model results were most similar to a joint GWAS combining multi-country and multi-breed data. First, Z-scores were estimated for each within-country GWAS as:

$${Z}_{ik}={\Phi }^{-1}\left(1-\frac{{p}_{ik}}{2}\right)\times {\Delta }_{ik},$$

where ${Z}_{ik}$ was the within-country Z-score for variant $i$ in GWAS $k$, $\Phi$ the standard normal cumulative distribution function, ${p}_{ik}$ the p-value estimated in the GWAS $k$, and ${\Delta }_{ik}$ the direction of effect in GWAS $k$. The Z-score for the meta-analysis for variant $i$ was equal to:

$${Z}_{i}=\frac{{\sum }_{k}{z}_{ik}{w}_{k}}{\sqrt{\sum_{k}{w}_{k}^{2}}},$$

where ${w}_{k}$ was the square root of the number of individuals in GWAS $k$. Finally, the p-value for variant $i$ in the meta-analysis was estimated as:

$${p}_{i}=2\Phi \left(-\left|{Z}_{i}\right|\right).$$

To define QTL, we used a threshold of 10^–6^ and considered that all the variants with a p-value below this threshold were significant. The false discovery rate (FDR) corresponding to a p-value threshold of 10^–6^ was estimated as $FDR=(nVariants \times {10}^{-6})/nSignificant$, where $nVariants$ is the total number of variants included in the meta-GWAS and $nSignificant$ the number of significant variants. Subsequently, significant variants located within 1 Mb of each other were grouped in the same QTL interval. In total, we repeated the within-country GWAS and meta-GWAS six times: once using the full dataset (to maximise power; used for QTL detection and genetic parameters), and then once for each of the five reference populations (used for genomic prediction).

### Selection of sequence variants for genomic prediction

Sequence variants were selected using the following steps:

1. All variants with the same direction of effect in both the AUS and NZL GWAS were selected.
2. Linkage disequilibrium (LD) pruning was performed in this selected variant set combining the genotypes of AUS and NZL animals in the reference population using the PLINK sofware [28] with an r^2^ threshold of 0.9, to avoid selecting redundant variants associated with the same QTL.
3. From the remaining variants, the 50,000 variants with the smallest p-value based on the meta-analyses were selected, to reduce the number of variants to that comparable with commonly used commercial SNP chips and to reduce computational demand. We refer to this set of variants as SEQ.

We selected six different sets of SEQ. The first set was done using the full dataset, and was only used to estimate genetic parameters. For genomic prediction, a new set of SEQ was selected for each fold, using only the reference population of that fold. Accuracies were then estimated for the validation population, which was not used for the selection of SEQ.

### Heritability and genetic correlation of MUN between Australia and New Zealand

Genetic parameters were estimated using the AIREMLF90 software [29]. The following bivariate model was used to estimate the heritability of MUN, and the genetic correlation between MUN in AUS and MUN in NZL, using either HD or SEQ variants:

$$\left[\begin{array}{c}{\mathbf{y}}_{\mathbf{A}\mathbf{U}\mathbf{S}}\\ {\mathbf{y}}_{\mathbf{N}\mathbf{Z}\mathbf{L}}\end{array}\right]=\left[\begin{array}{cc}{\mathbf{X}}_{\mathbf{A}\mathbf{U}\mathbf{S}}& \mathbf{0}\\ \mathbf{0}& {\mathbf{X}}_{\mathbf{N}\mathbf{Z}\mathbf{L}}\end{array}\right]\left[\begin{array}{c}{\mathbf{b}}_{\mathbf{A}\mathbf{U}\mathbf{S}}\\ {\mathbf{b}}_{\mathbf{N}\mathbf{Z}\mathbf{L}}\end{array}\right]+\left[\begin{array}{cc}{\mathbf{Z}}_{\mathbf{A}\mathbf{U}\mathbf{S}}& \mathbf{0}\\ \mathbf{0}& {\mathbf{Z}}_{\mathbf{N}\mathbf{Z}\mathbf{L}}\end{array}\right]\left[\begin{array}{c}{\mathbf{a}}_{\mathbf{A}\mathbf{U}\mathbf{S}}\\ {\mathbf{a}}_{\mathbf{N}\mathbf{Z}\mathbf{L}}\end{array}\right]+\left[\begin{array}{c}{\mathbf{e}}_{\mathbf{A}\mathbf{U}\mathbf{S}}\\ {\mathbf{e}}_{\mathbf{N}\mathbf{Z}\mathbf{L}}\end{array}\right],$$

where ${\mathbf{y}}_{\mathbf{A}\mathbf{U}\mathbf{S}}$ and ${\mathbf{y}}_{\mathbf{N}\mathbf{Z}\mathbf{L}}$ are vectors of MUN phenotypes for AUS and NZL cows, ${\mathbf{X}}_{\mathbf{A}\mathbf{U}\mathbf{S}}$ and ${\mathbf{X}}_{\mathbf{N}\mathbf{Z}\mathbf{L}}$ are matrices linking AUS and NZL cows to the fixed effect vectors ${\mathbf{b}}_{\mathbf{A}\mathbf{U}\mathbf{S}}$ and ${\mathbf{b}}_{\mathbf{N}\mathbf{Z}\mathbf{L}}$ with fixed effects (test month and herd-year-season) and linear covariates (days in milk, age, PC1), ${\mathbf{Z}}_{\mathbf{A}\mathbf{U}\mathbf{S}}$ and ${\mathbf{Z}}_{\mathbf{N}\mathbf{Z}\mathbf{L}}$ are incidence matrices linking AUS and NZL cows to the vectors of additive breeding values ${\mathbf{a}}_{\mathbf{A}\mathbf{U}\mathbf{S}}$ and ${\mathbf{a}}_{\mathbf{N}\mathbf{Z}\mathbf{L}}$ distributed as $\left(\frac{{a}_{AUS}}{{a}_{NZL}}\right)\sim N\left(\mathbf{0},\mathbf{G} \otimes {\mathbf{V}}_{a}\right)$, and ${\mathbf{e}}_{\mathbf{A}\mathbf{U}\mathbf{S}}$ and ${\mathbf{e}}_{\mathbf{N}\mathbf{Z}\mathbf{L}}$ are vectors of random residuals distributed as $\left(\frac{{e}_{AUS}}{{e}_{NZL}}\right)\sim N(\mathbf{0},\mathbf{G} \otimes {\mathbf{V}}_{e})$, $\mathbf{G}$ is a genomic relationship matrix constructed following VanRaden’s method 1 [30], $${\mathbf{V}}_{a}= \left[\begin{array}{cc}{\sigma }_{{a}_{AUS}}^{2}& {\sigma }_{{a}_{AUS,NZL}}\\ {\sigma }_{{a}_{AUS,NZL}}& {\sigma }_{{a}_{NZL}}^{2}\end{array}\right]$$, ${\sigma }_{{a}_{AUS}}^{2}$ the additive genetic variance in AUS, ${\sigma }_{{a}_{AUS,NZL}}$ the additive genetic covariance between AUS and NZL, ${\sigma }_{{a}_{NZL}}^{2}$ the additive genetic variance in NZL, $${\mathbf{V}}_{e}= \left[\begin{array}{cc}{\sigma }_{{e}_{AUS}}^{2}& 0\\ 0& {\sigma }_{{e}_{NZL}}^{2}\end{array}\right]$$, ${\sigma }_{{e}_{AUS}}^{2}$ the residual variance in AUS, and ${\sigma }_{{e}_{NZL}}^{2}$ the residual variance in NZL. In the bivariate analysis, we used the algorithm for proven and young (APY) [31] as implemented in AIREMLF90 [29] to stay within the memory limit of the software. We first estimated the number of cows required to explain 95% of the variance of $\mathbf{G}$, and then randomly selected this number of cows as core animals.

### Genomic prediction

We estimated the accuracy of genomic prediction for the following scenarios:

- WC_HD = within-country reference population, using HD variants,
- WC_SEQ = within-country reference population, using SEQ variants,
- MC_HD = multi-country reference population, using HD variants,
- MC_SEQ = multi-country reference population, using SEQ variants.

The same bivariate model described above was used to train the five reference populations and predict genomic estimated breeding values (GEBV) for the animals in the validation populations. Genetic parameters were re-estimated for each of the scenarios. Then, prediction accuracy was calculated as the correlation between the GEBV of the validation populations and their phenotypes corrected for fixed effects. Prediction accuracies were first obtained for each of the five validation populations, and subsequently the average of the five correlations was calculated. The bias was estimated as the regression coefficient of phenotypes corrected for fixed effects on GEBV.

## Results

### Principal component analysis and relationships between breeds

**Fig. 1:**
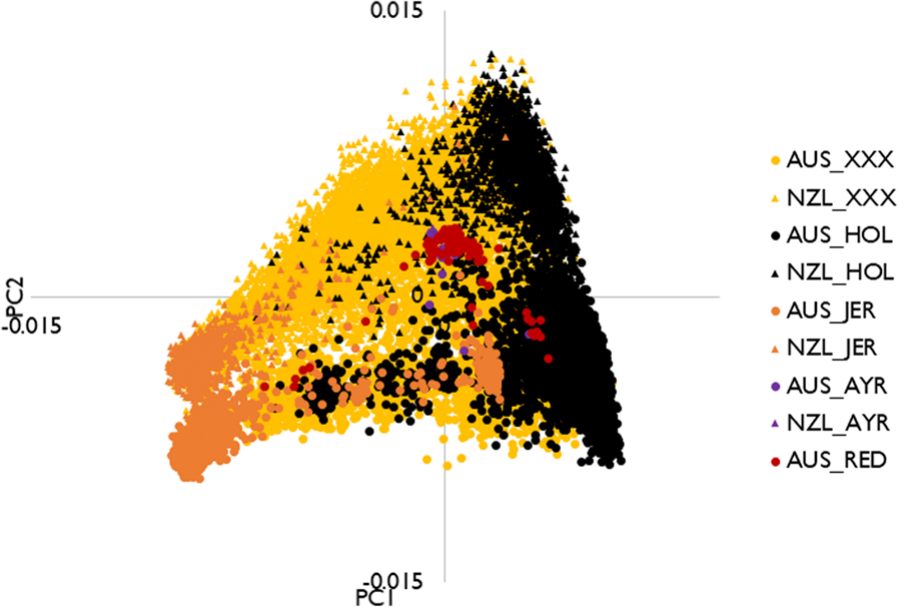
First (PC1) and second (PC2) principal components. *AUS_XXX* Australian crossbred, *NZL_XXX* New Zealand crossbred, *AUS_HOL* Australian Holstein, *NZL_HOL* New Zealand Holstein, *AUS_JER* Australian Jersey, *NZL_JER* New Zealand Jersey, *AUS_AYR* Australian Ayrshire, *NZL_AYR* New Zealand Ayrshire, *AUS_RED* Australian Red

Figure 1 shows the first two principal components, PC1 and PC2. Visually, PC1 separated Holsteins and Jerseys, with the crossbreds, Australian Reds and Ayrshires, being spread between the Holstein and Jersey clusters. PC1 and PC2 explained 6.6% and 1.4% of the genetic relationship matrix (GRM) variance, respectively.

The average within- and between-population genomic relationships of the GRM constructed with either the HD or SEQ variants were similar (see Additional file 1: Table S1). Within populations, the average genomic relationships were weakest in crossbreds (0.02 in AUS and NZL using either HD or SEQ variants) and strongest in Ayrshires (ranging from 0.18 in AUS Ayshire using HD to 0.38 in NZL Ayrshire using SEQ variants). The average genomic relationships between AUS and NZ were low for Holsteins (0.01 using either HD or SEQ variants) and crossbreds (− 0.01 using either HD or SEQ variants), but higher for Jerseys (0.16 using either HD or SEQ variants) and Ayrshires (0.14 using HD and 0.20 using SEQ variants).

### GWAS for QTL detection

**Fig. 2:**
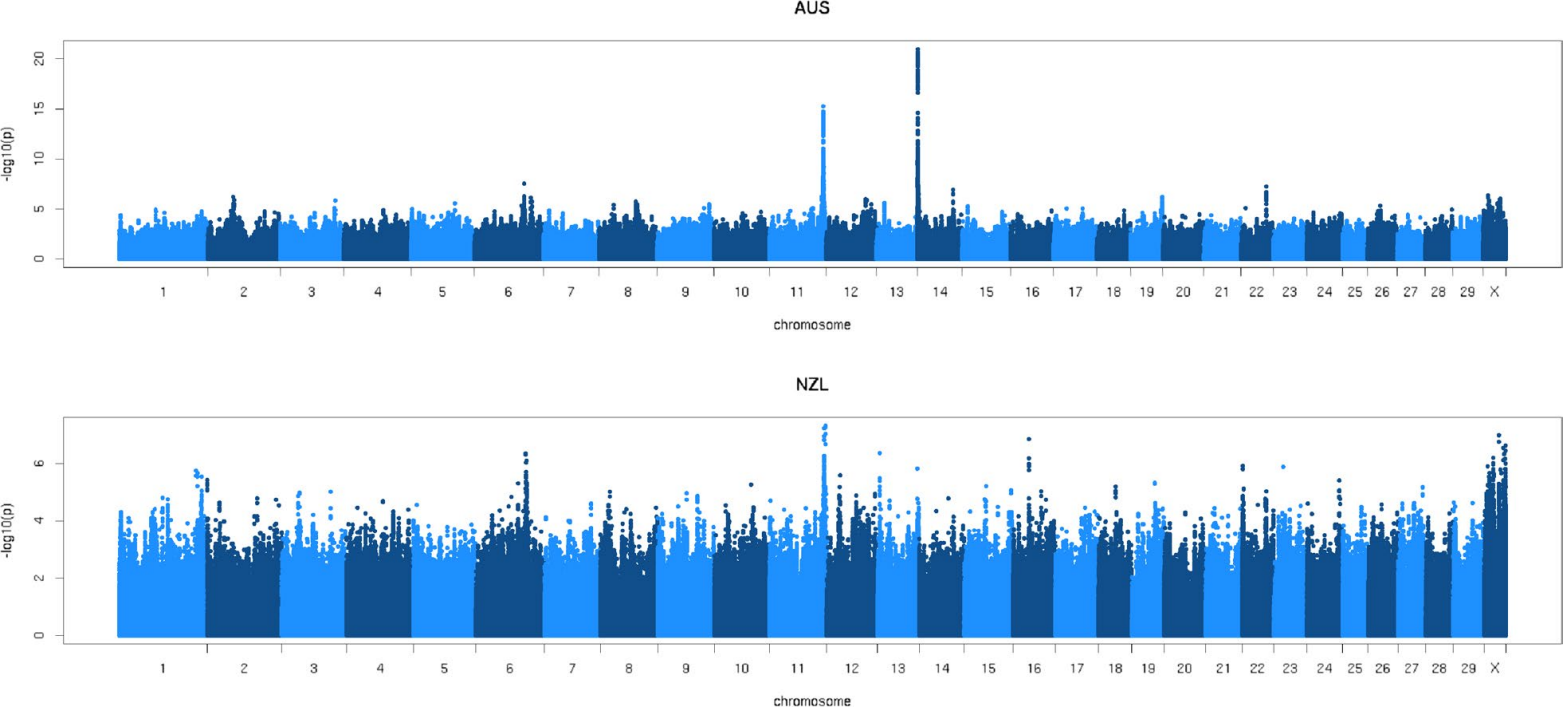
Manhattan plot of within-country GWAS for Australia and New Zealand

**Fig. 3:**
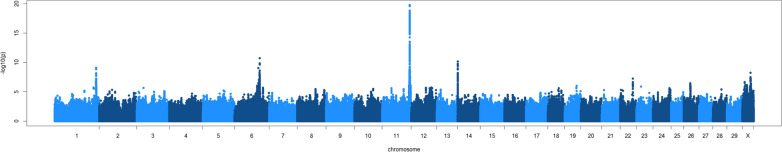
Manhattan plot of Australian–New Zealand meta-GWAS

Figure 2 compares the AUS GWAS with the NZL GWAS. Both GWAS revealed a peak on chromosome 11, with the most significant variant located at 103,271,858 bp in the AUS GWAS (p = 5.4 × 10^–16^), and 105,135,506 bp (p = 4.8 × 10^–8^) in the NZL GWAS. However, the peak detected with the AUS GWAS on chromosome 14 (top variant at 631,698 bp, p = 1.2 × 10^–21^) was not present in the NZL GWAS. The meta-GWAS (Fig. 3), combining the AUS and NZL GWAS, showed peaks with a p-value ≤ 10^–6^ on chromosomes 1, 6, 11, 14, 19, 22, 26 and the X chromosome, with the most significant variants located on chromosomes 11, 6 and 14. In total, there were 1244 significant variants detected in the meta-GWAS, corresponding to a FDR of 0.01. Table 2 provides more details on the QTL detected in the meta-analysis. The most significant variant was a synonymous variant located in the *glycosyltransferase 6 domain containing 1* (*GLT6D1*) gene, located at 103,271,858 bp on chromosome 11. This variant had p-values of 1.7 × 10^–20^, 5.4 × 10^–16^ and 8.8 × 10^–7^ in the meta-analysis, AUS GWAS and NZL GWAS, respectively. There were many significant variants on chromosome 11, of which 720 were located between 100,851,568 and 104,180,090 bp, encompassing multiple genes including *progestagen-associated endometrial protein* (*PAEP*). The QTL region detected on chromosome 6 contained 225 significant variants and spanned several genes, including *casein alpha S1* (*CSN1S1*), *casein alpha S2* (*CSN1S2*), *casein beta* (*CSN2*) and *casein kappa* (*CSN3*). The most significant variant was an intergenic variant located at 85,635,311 bp with a p-value of 1.9 × 10^–11^, 5.8 × 10^–7^ and 7.3 × 10^–6^ in the meta-analysis, AUS GWAS and NZL GWAS, respectively. The third most significant QTL interval was located between 520,394 and 633,066 bp on chromosome 14. The most significant variant, at 525,863 bp, was a downstream variant in ENSBTAG00000053637 with p-values of 6.6 × 10^–11^, 9.3 × 10^–19^ and 9.3 × 10^–1^ in the meta-analysis, AUS GWAS and NZL GWAS, respectively. The QTL region on chromosome 14 contained 102 significant variants and included the *diacylglycerol O-acyltransferase homolog 1* (*DGAT1*) gene. Additional file 2: Table S2 lists all the genes that are associated with significant variants in the detected QTL regions.

**Table 2:** QTL detected in the meta-analysis for MUN

Chr	Pos (bp)	p_meta_	p_AUS_	p_NZL_	Annotation	Genes	Start (bp)	End (bp)	N
1	148,821,034	8.2 × 10^–10^	2.1 × 10^–5^	8.7 × 10^–6^	Intergenic	*CLDN14-RF00001*	148,009,827	149,831,239	62
6	77,564,636	8.7 × 10^–7^	1.1 × 10^–3^	2.0 × 10^–4^	Intron	*ADGRL3*	77,558,465	77,564,636	2
6	81,099,934	9.3 × 10^–10^	5.0 × 10^–6^	4.5 × 10^–5^	Intron	*EPHA5*	81,099,934	84,329,940	35
6	85,635,311	1.9 × 10^–11^	5.8 × 10^–7^	7.3 × 10^–6^	Intergenic	*ODAM-CSN3*	85,389,494	86,548,307	225
11	103,271,858	1.7 × 10^–20^	5.4 × 10^–16^	8.8 × 10^–7^	Synonymous	*GLT6D1*	100,851,568	104,180,090	720
11	105,520,434	9.6 × 10^–7^	3.8 × 10^–4^	7.4 × 10^–4^	Intergenic	*ENSBTAG00000054425-OLFM1*	105,520,433	105,520,434	2
14	525,863	6.6 × 10^–11^	9.3 × 10^–19^	9.3 × 10^–1^	Downstream	*ENSBTAG00000053637*	520,394	633,066	102
19	47,763,063	9.5 × 10^–7^	6.5 × 10^–4^	4.2 × 10^–4^	Downstream	*TANC2*	47,763,063	47,763,063	1
22	47,184,592	5.8 × 10^–8^	5.8 × 10^–8^	–	Intron	*CACNA1D*	47,163,588	47,286,336	36
26	21,278,969	3.6 × 10^–7^	1.4 × 10^–4^	7.0 × 10^–4^	3ʹUTR	*SCD*	21,277,195	21,284,652	10
X	16,376,624	2.2 × 10^–7^	7.2 × 10^–7^	2.2 × 10^–2^	Intergenic	*MBNL3-HS6ST2*	16,366,703	16,376,624	6
X	31,227,651	6.4 × 10^–7^	1.3 × 10^–3^	1.2 × 10^–4^	Intron	*AFF2*	31,175,135	31,227,651	12
X	54,324,305	7.2 × 10^–7^	1.6 × 10^–4^	1.3 × 10^–3^	Intergenic	*ENSBTAG00000009457-ENSBTAG00000045756*	54,324,305	54,324,305	1
X	106,058,470	5.7 × 10^–9^	2.9 × 10^–6^	4.3 × 10^–4^	Upstream	*PRRG1*	105,580,018	108,413,114	30

*MUN* milk urea nitrogen, *chr* chromosome, *pos* position of most significant variant associated with the QTL, *bp* base pair according to the ARS-UCD1.2 annotation, *p*_*meta*_ p-value in meta-analysis, *p*_*AUS*_ p-value in Australian GWAS, *p*_*NZL*_ p-value in New Zealand GWAS, annotation the annotation of the most significant variant, *genes* the gene in which the most significant region is located or the genes between which an intergenic variant is located, *start and end* start and end of QTL interval, *N* number of variants with p-values ≤ 10^–6^ within the QTL interval

### Selection of sequence variants

**Fig. 4:**
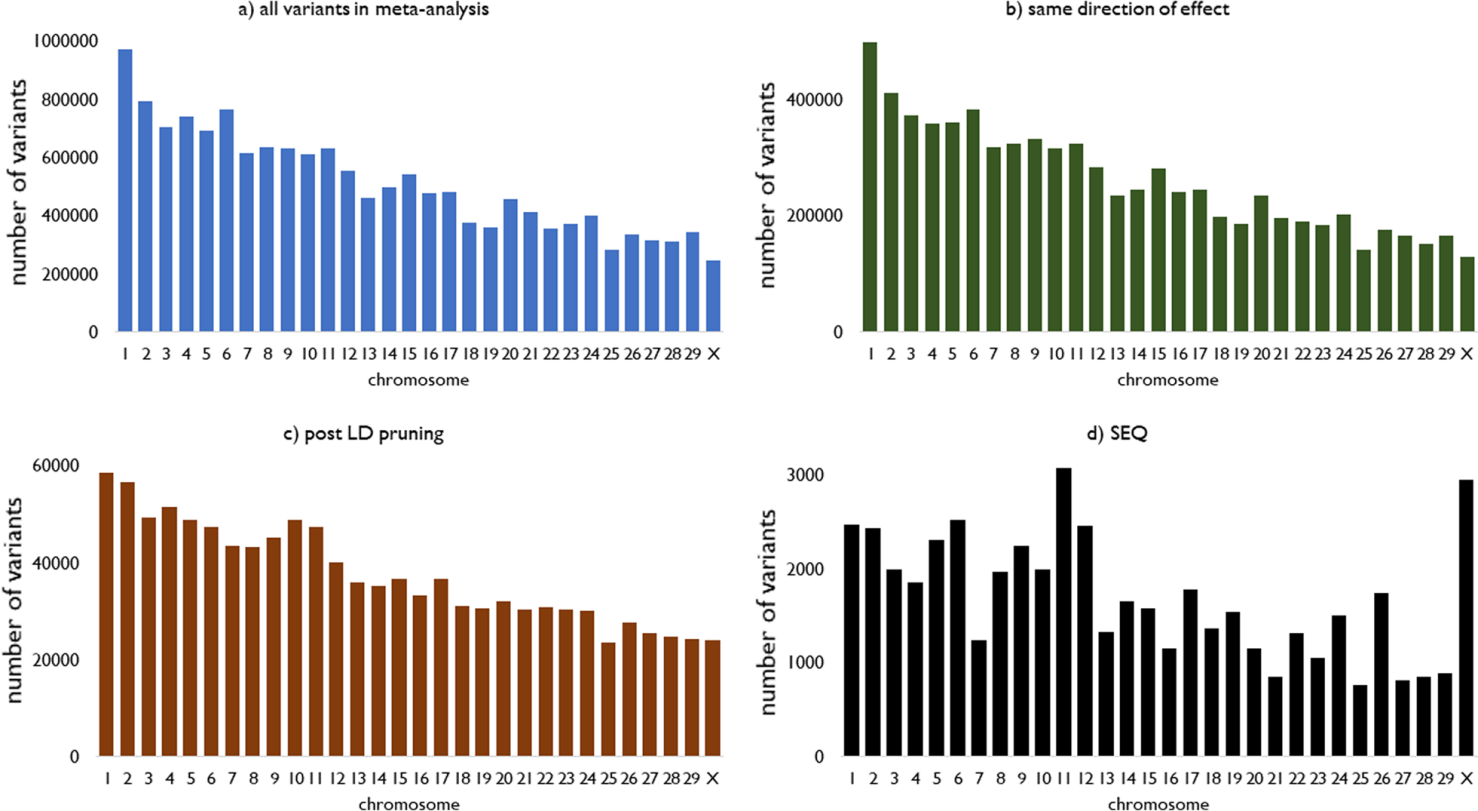
Number of variants per chromosome for different sets of variants. **a** shows all variants used in the meta-analysis, **b** variants in **a** that had the same direction of effect in the Australian and New Zealand GWAS, **c** variants in **b** after pruning for linkage disequilibrium (LD) and **d** the 50,000 variants in **c** that had the smallest p-value in the meta-analysis

Figure 4 shows the number of variants per chromosome selected during each of the selection steps, using the full dataset to select SEQ variants. Out of the 15,081,121 variants included in the meta-analysis and within-country GWAS, 7,703,884 (51%) had the same direction of effect in AUS and NZL. After LD pruning, 1,106,212 variants remained, with more variants located on the larger chromosomes. SEQ variants were spread over all the chromosomes, with between 738 and 3047 variants per chromosome. Chromosomes 11, X and 6 contained the largest numbers of variants, with 3047, 2920 and 2494 variants, respectively. The p-values of the selected variants ranged from 2.3 × 10^–17^ to 0.019.

Selecting variants within the reference populations designed for the cross-validation process resulted in five different sets of 50,000 variants each. Additional file 3: Figure S1 shows the overlap between these sets of selected sequence variants. In total, 16,041 selected variants were common to the five reference populations, and between 8404 to 8897 variants were unique to each of the reference populations.

### Genetic parameters of SEQ and HD variants

Table 3 shows the heritabilities of MUN using the different sets of variants and the genetic correlation between MUN in AUS and MUN in NZL. Heritabilities were higher when only the SEQ variants were used than when HD variants were used, with heritabilities of 0.26 ± 0.01 (AUS) and 0.44 ± 0.01 (NZL) using the SEQ variants and 0.11 ± 0.01 (AUS) and 0.30 ± 0.01 (NZL) using the HD variants. The genetic correlation between MUN in AUS and in NZL increased substantially using the SEQ variants, from 0.27 ± 0.07 using the HD variants to 0.95 ± 0.02 using the SEQ variants. Similar genetic correlations were obtained in the cross-validation folds, ranging from 0.30 to 0.36 for the HD variants and from 0.93 to 1.00 for the SEQ variants.

**Table 3:** Heritability of and genetic correlation between MUN in Australia and in New Zealand (${{r}}_{{g}},{{A}}{{U}}{{S}}-{{N}}{{Z}}{{L}}$) using high-density and sequence variants

Parameter	HD	SEQ
${h}_{AUS}^{2}$	0.11 ± 0.01	0.26 ± 0.01
${h}_{NZL}^{2}$	0.30 ± 0.01	0.44 ± 0.01
${r}_{g,AUS-NZL}$	0.27 ± 0.07	0.95 ± 0.02

Heritabilities and genetic correlations were estimated using either the routine high density bovine SNP chip (HD) or 50,000 selected sequence variants (SEQ). Heritabilities were estimated using Australian (${h}_{AUS}^{2}$) or New Zealand (${h}_{NZL}^{2}$) data. Estimates are followed by their standard error

In the bivariate analyses to estimate the genetic correlation between MUN in Australia and MUN in New Zealand, a core set of 9157 animals was required to explain 95% of the variance of $\mathbf{G}$. Additional file 4: Table S3 provides the breed and country composition of the animals that were selected for the core set.

### Genomic prediction accuracy and bias

**Fig. 5:**
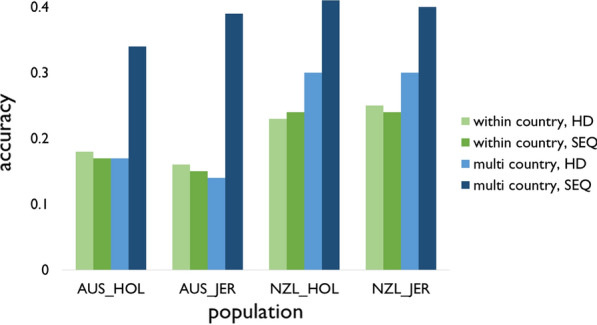
Genomic prediction accuracy comparing reference populations and sets of variants. *WC_HD* within country reference population, using variants on the Illumina Bovine HD BeadChip (HD), *WC_SEQ* within country reference population, using selected sequence variants (SEQ), *MC_HD* multi country reference population, using HD variants, *MC_SEQ* multi country reference population, using SEQ variants, *AUS_HOL* Australian Holstein cows, *AUS_JER* Australian Jersey cows, *NZL_HOL* New Zealand Holstein cows, *NZL_JER* New Zealand Jersey cows

Figure 5 shows the genomic prediction accuracies averaged over the five folds. The accuracies for each fold are in Additional file 5: Table S4. Using HD variants, the multi-country reference population increased the prediction accuracy in the NZL cows compared to the within-country reference population, from 0.23 to 0.30 for NZL Holstein and from 0.25 to 0.30 for NZL Jersey. For the AUS validation cows, the multi-country reference population yielded slightly lower accuracies than the within-country reference population: the accuracy decreased from 0.18 to 0.17 in AUS Holstein, and from 0.16 to 0.14 in AUS Jersey.

Using the SEQ variants instead of the HD variants resulted only in minor changes in accuracy using the within-country reference populations. However, the SEQ variants did substantially increase the accuracy in all populations using the multi-country reference population. Averaged across populations, accuracies obtained with the SEQ variants were equal to 0.20 using a within-country reference population and 0.39 using a multi-country reference population.

**Fig. 6:**
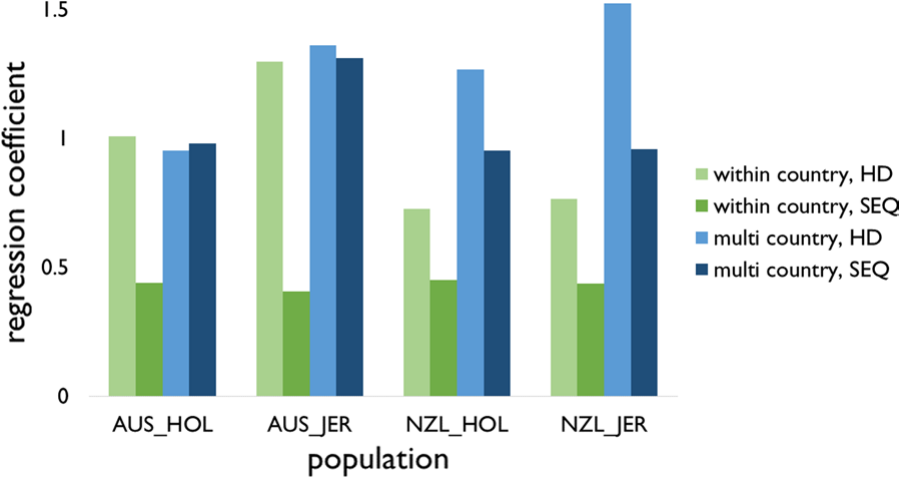
Genomic prediction bias comparing reference populations and sets of variants. *WC_HD* within country reference population, using variants on the Illumina Bovine HD BeadChip (HD), *WC_SEQ* within country reference population, using selected sequence variants (SEQ), *MC_HD* multi country reference population, using HD variants, *MC_SEQ* multi country reference population, using SEQ variants, *AUS_HOL* Australian Holstein cows, *AUS_JER* Australian Jersey cows, *NZL_HOL* New Zealand Holstein cows, *NZL_JER* New Zealand Jersey cows

The SEQ variants increased the bias substantially when a within-country reference population was used (Fig. 6). For example, the regression coefficient for within-country prediction in AUS Holstein was 1.01 with the HD variants and 0.44 with the SEQ variants. However, for multi-country prediction the biases using the SEQ variants were similar to or smaller than those obtained with the HD variants. For multi-country prediction in NZL Holstein, a regression coefficient of 1.27 was obtained using the HD variants which decreased to 0.95 when using the SEQ variants.

## Discussion

We detected several QTL for MUN and showed how sequence variants that were selected based on GWAS for MUN can be used to substantially increase the prediction accuracy for MUN in AUS and NZL when using a multi-country reference population.

### GWAS on AUS and NZL cows

Interestingly, while several peaks were present in both the AUS and NZL GWAS, the major peak in the AUS GWAS that was on chromosome 14 was not present in the NZL GWAS. The relatively low genetic correlations between MUN in AUS and in NZL [5] indicate that MUN in AUS and MUN in NZL may not, from a genetic perspective, be considered as the same trait. Hence, differences in GWAS were expected. The peak on chromosome 14 included variants that are located in the *DGAT1* gene, a major QTL associated with fat yield in dairy cattle [32]. MUN in AUS has a significant correlation with fat yield, while this is not the case for MUN in NZL [5], which may explain why the peak on chromosome 14 was present in the AUS but not in the NZL GWAS.

### QTL detected for MUN

We detected 14 QTL located on eight chromosomes with p-values ≤ 10^–6^ in the meta-analysis. The largest three QTL intervals, on chromosomes 6, 11 and 14, all included well-known QTL associated with milk traits in dairy cattle [32–35]. The most significant QTL interval detected on chromosome 6, between 85,389,494 and 86,548,307 bp, overlaps with a QTL detected for MUN by Ariyarathne et al. [13]. While the most significant variant in this QTL was an intergenic variant, the interval included the casein genes *CSN1S1*, *CSN1S2*, *CSN2* and *CSN3*, which are associated with milk production traits [33, 34]. The most significant QTL interval on chromosome 11 overlapped with another QTL reported by Ariyarathne et al. [13]. This interval was located between 100,851,568 and 104,180,090 bp, with a synonymous variant located in the *GLT6D1* gene as the most significant variant. *GLT6D1* is associated with periodontitis in humans [36]. The QTL interval also included *PAEP*, a gene that is assumed to underlie a well-known QTL associated with milk production traits in dairy cattle [35]. Another gene in this QTL interval is *alpha 1-3-N-acetylgalactosaminyltransferase and alpha 1-3-galactosyltransferase* (*ABO*), which determines blood type in humans [37] and has been associated with protein yield in dairy cattle [38]. van den Berg et al. [39] reported an overlap between an eQTL for *ABO* and a QTL for protein yield and protein percentage in dairy cattle. The most significant variant on chromosome 14 was a downstream variant in *ENSBTAG00000053637*, and that QTL interval included *DGAT1*, a major QTL for milk production traits in dairy cattle [32]. The most significant variant on chromosome 26 was located in the 3’UTR region of the *stearoyl-CoA desaturase* (*SCD*) gene that has previously been associated with MUN [15] and milk fatty acid composition [40].

### Selection of sequence variants and genomic prediction

Several previous studies have demonstrated that sequence variants selected from a GWAS can be used to improve the accuracy of genomic prediction [9–11], as we did for MUN. The strategy that we used to select SEQ variants was very similar to that used by Porto-Neto et al. [10], who selected the top 10% variants from 71,726 SNPs that had the same direction of effect in two beef cattle breeds and achieved substantial increases in the proportion of variance explained by the variants, in genetic correlations between populations and in genomic prediction accuracy. However, the sequence variants selected by Porto-Neto et al. [10] also resulted in an increase in bias using a multibreed reference population. MacLeod et al. [41] showed that using the same set of animals for the selection of sequence variants and the reference set used for genomic prediction can lead to increases in bias. In our study, the SEQ variants helped to achieve increases in the proportion of variance explained, in genetic correlations between populations and in genomic prediction accuracy for multi country prediction, without increasing the bias. Excluding the validation animals from the GWAS likely helped to limit the bias. However, using the SEQ variants did increase bias when used for within-country prediction. Given that the variants in SEQ were selected for the specific purpose of multi-country prediction and not for within-country prediction, the increased bias observed for within-country prediction may not be a concern.

Both the heritability and genetic correlation between AUS and NZL were substantially higher with the SEQ than the HD variants. A possible explanation for this observation could be that the SEQ variants may be closer to the causal mutations for MUN than the HD variants, resulting in a larger proportion of the genetic variance being captured by the SEQ variants than by the HD variants. Alternatively, several studies have shown that using genomic markers to estimate heritability can lead to upward biased estimates compared to heritabilities estimated using pedigree data [42, 43]. The observed increase in genetic correlation using SEQ variants may reflect that when prediction markers are closer to the causal mutations, the probability that LD is conserved between populations increases, resulting in a higher genetic correlation and prediction accuracy. However, estimates of the genetic correlation using genomic data may be biased, especially when estimating genetic correlations across populations [44]. However, Wientjes et al. [44] found that biases in genetic correlations across populations were mostly downward. Furthermore, selecting variants with the same direction of effect in AUS and NZL probably caused an upward bias in the estimate of the genetic correlation. Hence, while the increased heritability and genetic correlation using SEQ variants may represent useful indicators for the increased prediction accuracy, caution should be taken when interpreting genomic parameters based on genomic markers.

While the genetic correlation between populations increased using SEQ variants, the genomic relationships in the SEQ GRM were largely similar using either the HD or SEQ variants. Larger differences were observed for Ayrshires and Australian Reds, but their small datasize makes it difficult to interpret these differences, and exclusion of Australian Reds and Ayrshires in the genomic prediction analyses did not have a substantial impact on the prediction accuracy using either HD or SEQ variants (differences in accuracy < 0.01; results not shown). Interestingly, the genomic relationships between AUS and NZL populations indicated that while the genetic links between AUS and NZL Holsteins and crossbreds are small, the AUS and NZL Jerseys showed stronger genomic relationships.

Although the results using the SEQ variants for multi-country prediction are promising, we tested our approach only for the specific purpose of combining AUS and NZL data for MUN. Hence, results may be different for other traits and when combining different populations, such as multiple breeds, and further testing is required to investigate whether the strategy used to select SEQ variants can help increase prediction accuracy for a wider range of traits and populations.

The discovery populations used to select the SEQ variants were the same as the reference populations used for genomic prediction, and reference and validation populations were highly related due to the design of the cross-validation scheme and the available data for this study. For example, it was possible for half sisters to be present in both the reference and validation populations in the same fold, which likely resulted in higher accuracies compared to cross-validation designs where reference and validation populations are less related. The promising results that we obtained in terms of increased genetic correlation and genomic prediction using a meta-GWAS and a joint reference population of AUS and NZL cows need to be assessed when validation cows are selected based on date of birth and when validation cows that are less related to the reference population are used. Furthermore, it will be useful to confirm our results by selecting variants from a dataset that does not include animals in the reference population used for genomic prediction as recommended in [41]. We did not test different discovery, reference and validation population designs because of the relatively small data size for MUN, especially for pure NZL HOL and JER cows.

## Conclusions

Our results demonstrate how, in spite of the low genetic correlation between MUN measured in two countries using a standard SNP chip, sharing data from different sources internationally can be beneficial to increase the power of a GWAS and to increase the accuracy of genomic prediction using a multi-country reference population and sequence variants selected based on GWAS. Our approach of selecting sequence variants to increase prediction accuracy may be useful for multi-breed and multi-country prediction, but first this needs to be tested on a wider range of traits and populations.

## Supplementary Information

**Additional file 1: Table S1.** Genomic relationships using HD and SEQ variants. Average genomic relationships within (diagonal) and between populations (below the diagonal) of genomic relationship matrices constructed using high-density (HD) or selected sequence (SEQ) variants; AUS = Australia, NZL = New Zealand.

**Additional file 2: Table S2.** Genes located within QTL regions. chr = chromosome, start and end = start and end of QTL region in base pair (bp) on the ARS-UCD1.2 annotation, genes = list of genes located within QTL region.

**Additional file 3: Figure S1.** Overlap between sets of sequence variants selected in cross-validation folds. Ref1, ref2, ref3, ref4 and ref5 are the five reference populations that were used to select five sets of 50,000 sequence variants with the same direction of effect for MUN in AUS and NZL that were most significant in the meta-analysis for the GWAS five sets of variants, and the numbers represent the overlap between the different sets of variants.

**Additional file 4: Table S3.** Composition of the animals in the core set. Number of animals of each population included in the core set.

**Additional file 5: Table S4.** Genomic prediction accuracies for each of the five folds. validation = validation population, reference = reference validation, fold1-5 = prediction accuracy in fold 1–5, AUS_HOL = Australian Holstein, AUS_JER = Australian Jersey, NZL_HOL = New Zealand Holstein, NZL_JER = New Zealand Jersey, WC = within country reference population, MC = multi country reference population, HD = variants on the Illumina Bovine HD BeadChip, SEQ = selected sequence variants.

## Data Availability

The data used for this research are not publicly available.
