# Supplementary figures and images for: GWAS and genomic prediction of milk urea nitrogen in Australian and New Zealand dairy cattle
Source: Genet Sel Evol. 2022 Feb 19;54:15. doi: 10.1186/s12711-022-00707-9 (PMC8858489; doi:10.1186/s12711-022-00707-9)

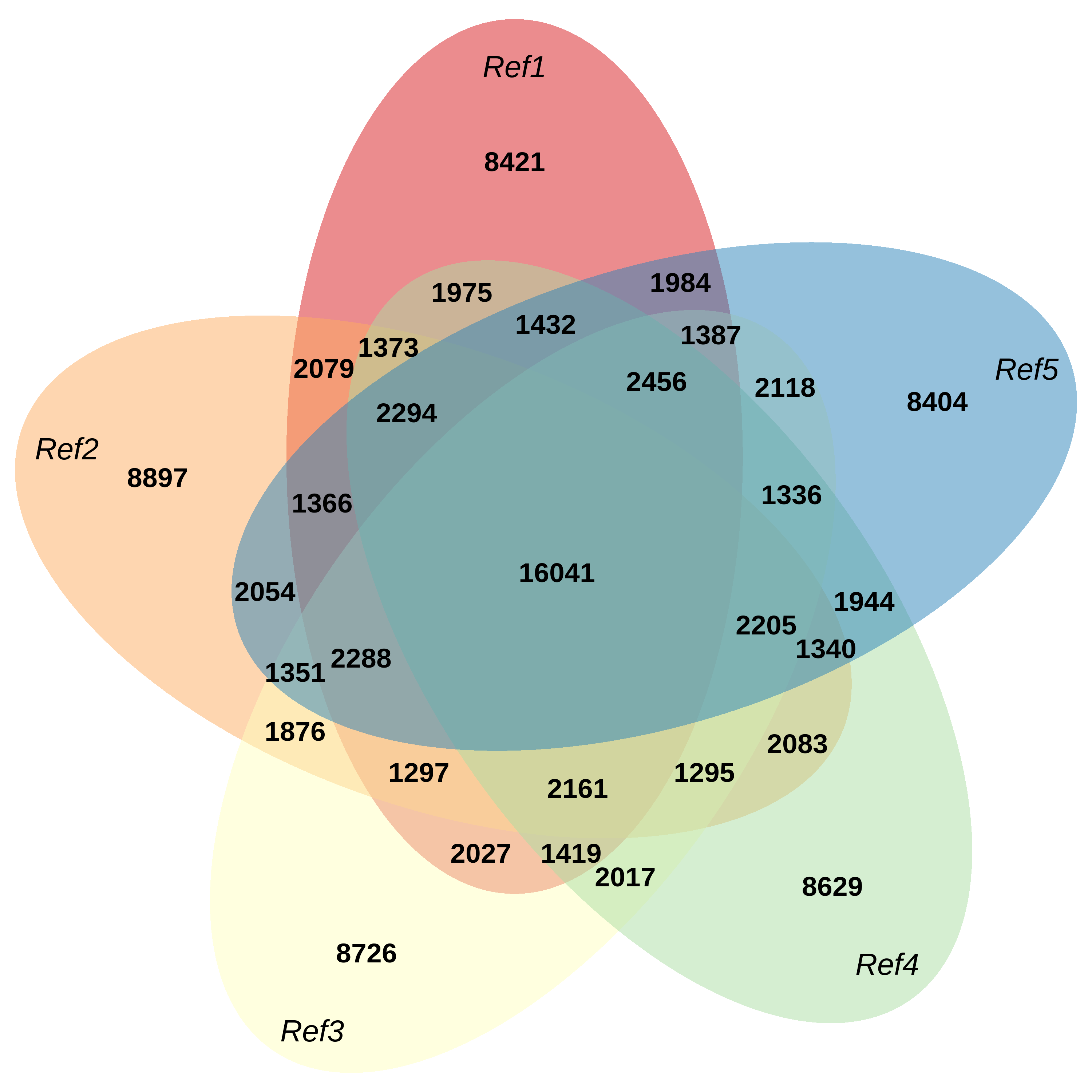

Supplement: Supplementary file 3 — Additional file 3: Figure S1. Overlap between sets of sequence variants selected in cross-validation folds. Ref1, ref2, ref3, ref4 and ref5 are the five reference populations that were used to select five sets of 50,000 sequence variants with the same direction of effect for MUN in AUS and NZL that were most significant in the meta-analysis for the GWAS five sets of variants, and the numbers represent the overlap between the different sets of variants. [file 12711_2022_707_MOESM3_ESM.png]
